# Commentary: Assessment of treatment effectiveness, follow-up results, and recurrence patterns in children with *Helicobacter pylori* infection

**DOI:** 10.3389/fped.2026.1955929

**Published:** 2026-09-03

**Authors:** Toshihiko Kakiuchi

**Affiliations:** Department of Pediatrics, Faculty of Medicine, Saga University, Saga, Japan

**Keywords:** children, *Helicobacter pylori*, recrudescence, recurrence, reinfection, urea breath test

## Introduction

This General Commentary refers to: Luo M, Wei D, Jin L, Wei Y. Assessment of treatment effectiveness, follow-up results, and recurrence patterns in children with *Helicobacter pylori* infection. Front Pediatr. 2026;14:1815432. doi:10.3389/fped.2026.1815432.

Luo et al. reported valuable longitudinal data regarding recurrence following *Helicobacter pylori* eradication therapy in children (1). Their study contributes important evidence to an area in which pediatric follow-up data remain limited. The reported recurrence rate is clinically relevant and deserves careful consideration. I believe, however, that the principal issue raised by this study is not the recurrence rate itself but the interpretation that can appropriately be drawn from the study design. I therefore wish to discuss how recurrence should be interpreted after eradication therapy.

In this Commentary, I use “recurrence” to denote the reappearance of *H. pylori* after an initially negative post-treatment test, irrespective of the underlying cause. “Recrudescence” and “reinfection” are used to describe the two principal biological mechanisms that may account for recurrence.

## Recurrence and reinfection are not interchangeable concepts

Clinical studies distinguish between an observed outcome and the biological mechanism responsible for that outcome. Following eradication therapy, recurrence is an observed clinical outcome, whereas recrudescence and reinfection are biological explanations for that outcome. These concepts should not be used interchangeably because they require different levels of evidence (2). Serial non-invasive testing can establish recurrence but cannot, by itself, determine the mechanism responsible for renewed positivity.

In the study by Luo et al., recurrence was operationally defined using serial 13C-urea breath testing after an initially negative post-treatment examination (1). This approach provides a practical assessment of post-treatment recurrence. However, eradication was assessed only four weeks after completion of therapy, whereas current ESPGHAN/NASPGHAN guidelines recommend assessment 6–8 weeks after treatment (3). Testing at four weeks may increase the risk of a false-negative result before residual organisms become detectable, so some later positive tests could represent recrudescence after an initially false-negative test rather than true new infection.

The accuracy of the initial negative UBT also depends on appropriate test preparation. Current pediatric guidance recommends withholding proton pump inhibitors for at least 2 weeks and antibiotics and bismuth salts for at least 4 weeks before testing (3). Luo et al. reported overnight fasting before UBT, but medication exposure immediately before follow-up testing was not detailed (1). Inadequate fasting can also reduce UBT sensitivity, particularly in children (4). These factors should therefore be considered when interpreting an initially negative post-treatment result.

**Figure 1:**
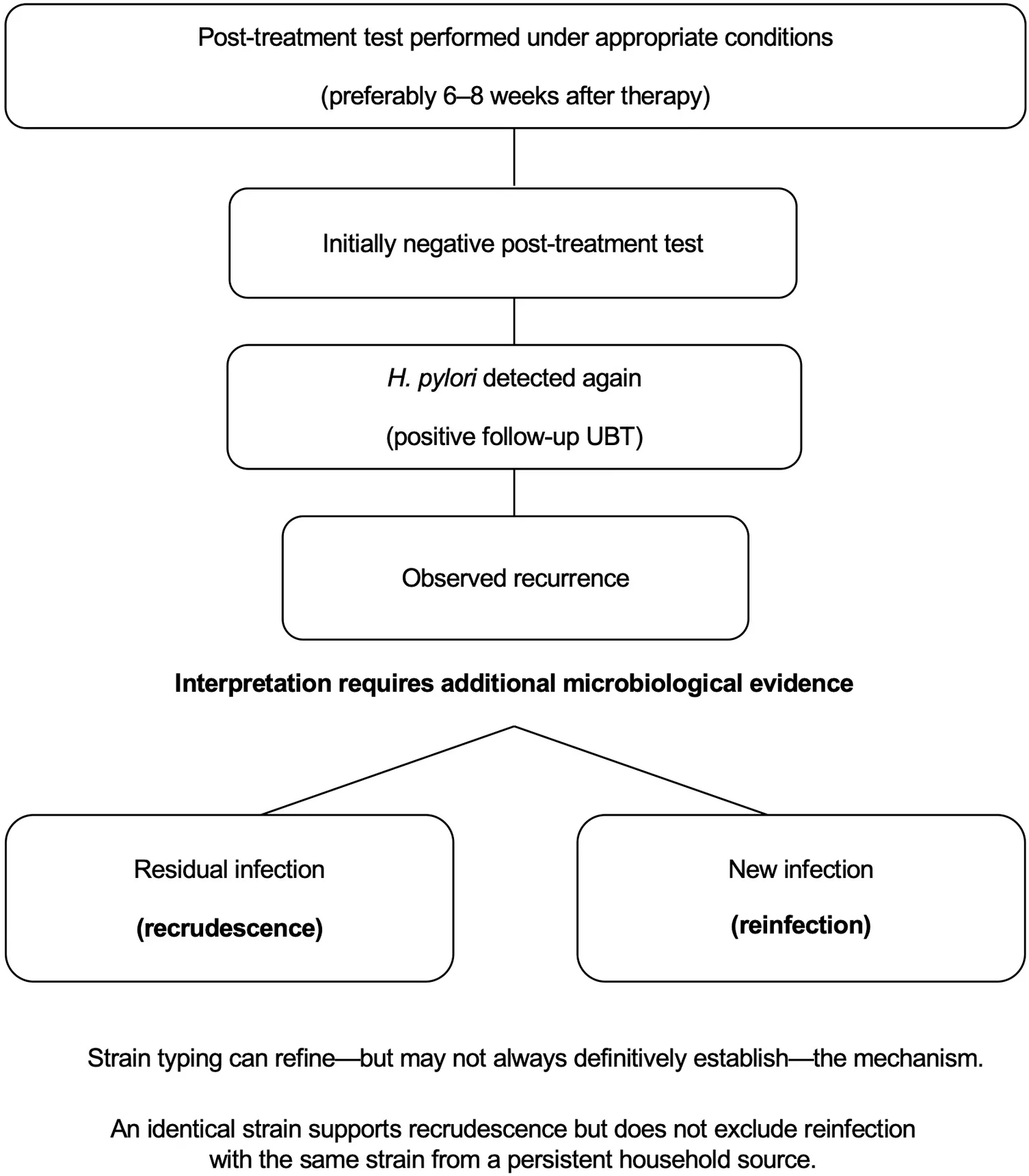
Conceptual framework for interpreting post-eradication recurrence. Serial UBT establishes recurrent positivity after an initially negative post-treatment test but does not identify its underlying biological mechanism. The initial negative result should itself be interpreted in light of recommended testing timing and preparation because false-negative results can occur. In the absence of bacterial strain typing or other molecular approaches, recurrent positivity cannot be attributed to recrudescence or reinfection. Even with strain typing, an identical strain supports but does not prove recrudescence because reinfection from a persistent household source with the same strain is possible. Accordingly, recurrent positivity is an observed clinical outcome, whereas the biological mechanism may remain uncertain. UBT, urea breath test.

Because bacterial strain typing or molecular characterization was not incorporated into the study design, the reported data cannot distinguish recrudescence from reinfection (Figure 1). Molecular comparison of pre- and post-treatment isolates could strengthen this distinction: detection of a genetically different strain would support reinfection, whereas recovery of the same strain would support recrudescence. However, even strain identity would not be conclusive, because a child could theoretically be reinfected with the same strain from a persistent household source. Thus, strain typing can refine, but not always definitively establish, the biological mechanism of recurrence.

Accurate interpretation of post-eradication recurrence is important because recurrence and reinfection have different implications for evaluating treatment efficacy and understanding the natural history of *H. pylori* infection. Misclassification of recurrent positivity as reinfection may underestimate treatment failure and overestimate the epidemiological burden of new infection. Conversely, interpreting all recurrent positivity as treatment failure may underestimate true reinfection in populations with ongoing exposure. Recent systematic reviews therefore distinguish recurrence, recrudescence, and reinfection as separate clinical outcomes (5).

Luo et al. observed 14 recurrent positive tests among 102 children initially classified as successfully eradicated (13.7%), a clinically notable proportion despite the modest number of recurrence events (1). Recurrence was discussed primarily in relation to environmental exposure and household transmission in younger children. These explanations are biologically plausible and consistent with previous literature (5). Nevertheless, because the study was not designed to distinguish reinfection from recrudescence, these interpretations are more appropriately regarded as hypotheses than as conclusions directly supported by the reported data. Explicit acknowledgement of this methodological limitation would further strengthen interpretation of the study findings.

Importantly, my intention is not to question the reported recurrence rate itself. Recent evidence suggests that recurrence following pediatric eradication therapy may be higher than previously recognized in some populations (5). Rather, my concern relates to interpretation. Failure to distinguish recurrence from its underlying mechanism may unintentionally lead readers to equate recurrence with reinfection, thereby influencing future systematic reviews, clinical interpretation, and subsequent pediatric *H. pylori* research.

## Discussion

Luo et al. provide valuable longitudinal evidence regarding recurrence following pediatric *Helicobacter pylori* eradication therapy. The principal methodological message arising from this study is the importance of distinguishing an observed clinical outcome from its biological explanation. The study demonstrates recurrent positivity after an initially negative post-treatment UBT; however, the four-week timing of the initial UBT, potential test-related false-negative factors, and the absence of strain-level characterization limit inference about the underlying mechanism. Clarifying these issues will strengthen interpretation of future post-eradication studies and help prevent overgeneralization of recurrence data in children.

## References

[1] Luo M Wei D Jin L Wei Y. Assessment of treatment effectiveness, follow-up results, and recurrence patterns in children with *Helicobacter pylori* infection. Front Pediatr. (2026) 14:1815432. 10.3389/fped.2026.1815432 42109477 PMC13149433

[2] Gisbert JP. The recurrence of *Helicobacter pylori* infection: incidence and variables influencing it. A critical review. Am J Gastroenterol. (2005) 100(9):2083–99. 10.1111/j.1572-0241.2005.50043.x 16128956

[3] Homan Mž Jones NL Bontems P Carroll MW Czinn SJ Gold BD. Updated joint ESPGHAN/NASPGHAN guidelines for management of *Helicobacter pylori* infection in children and adolescents 2023. J Pediatr Gastroenterol Nutr. (2024) 79(3):758–85. 10.1002/jpn3.12314 39148213

[4] Keller J Hammer HF Afolabi PR Benninga M Borrelli O Dominguez-Munoz E. European Guideline on indications, performance and clinical impact of 13C-breath tests in adult and pediatric patients: an EAGEN, ESNM, and ESPGHAN consensus, supported by EPC. United European Gastroenterol J. (2021) 9(5):598–625. 10.1002/ueg2.12099 34128346 PMC8259225

[5] Xu L Li X-T Ur-Rahman I Zhang C Qi Y-B Hu R-B. Global *H. pylori* recurrence, recrudescence, and re-infection status after successful eradication in pediatric patients: a systematic review and meta-analysis. J Gastroenterol. (2024) 59(8):668–81. 10.1007/s00535-024-02114-x 38814335

